# A review of deep learning-based reconstruction methods for accelerated MRI using spatiotemporal and multi-contrast redundancies

**DOI:** 10.1007/s13534-024-00425-9

**Published:** 2024-09-17

**Authors:** Seonghyuk Kim, HyunWook Park, Sung-Hong Park

**Affiliations:** 1 School of Electrical Engineering, Korea Advanced Institute of Science and Technology, Daejeon, Republic of Korea; 2 Department of Bio and Brain Engineering, Korea Advanced Institute of Science and Technology, 291 Daehak-ro, Yuseong-gu, Daejeon, 34141 Republic of Korea

**Keywords:** Accelerated MRI, Deep learning, Multi-contrast, Reconstruction, Redundancy, Spatiotemporal

## Abstract

Accelerated magnetic resonance imaging (MRI) has played an essential role in reducing data acquisition time for MRI. Acceleration can be achieved by acquiring fewer data points in k-space, which results in various artifacts in the image domain. Conventional reconstruction methods have resolved the artifacts by utilizing multi-coil information, but with limited robustness. Recently, numerous deep learning-based reconstruction methods have been developed, enabling outstanding reconstruction performances with higher acceleration. Advances in hardware and developments of specialized network architectures have produced such achievements. Besides, MRI signals contain various redundant information including multi-coil redundancy, multi-contrast redundancy, and spatiotemporal redundancy. Utilization of the redundant information combined with deep learning approaches allow not only higher acceleration, but also well-preserved details in the reconstructed images. Consequently, this review paper introduces the basic concepts of deep learning and conventional accelerated MRI reconstruction methods, followed by review of recent deep learning-based reconstruction methods that exploit various redundancies. Lastly, the paper concludes by discussing the challenges, limitations, and potential directions of future developments.

## Introduction

Magnetic resonance imaging (MRI) is a non-invasive imaging modality that is essential in many clinical practices. MRI offers numerous advantages over other tomographic imaging devices including computed tomography (CT) and positron emission tomography (PET). Different from CT or PET, ionizing radiation is not utilized for MRI data acquisition. Furthermore, MRI allows scanning at any plane of the body with excellent soft-tissue contrast. Therefore, MR images are frequently used by the clinicians for accurate diagnosis and disease management.

One renowned downside of MRI is the long data acquisition time. Accelerated MRI has been a successful option for reducing the scan time by acquiring smaller data points in the k-space. Direct reconstruction from undersampled k-space data yields various artifacts depending on the acquisition trajectory. Accordingly, numerous techniques and methods have been developed to reconstruct artifact-free images from undersampled k-space data. Examples of traditional reconstruction methods include parallel imaging (PI) [1, 2] and compressed sensing (CS) [3], both widely used in clinical practices.

Recently, deep learning has shown extraordinary developments. Implementation of neural networks with multiple layers of processing units allowed the learning of complex patterns in a large amount of data [4]. Application of deep learning has achieved significant advancements in medical imaging fields including medical image classification [5–7], segmentation [8–10], anomaly detection [11–13], and reconstruction [14–16]. The application of deep learning to accelerated MRI was first introduced using a large number of training data [17–19]. Since then, a substantial amount of deep learning approaches has been developed for accelerated MR image reconstruction [20–23].

Deep learning-based reconstruction methods typically exploit the relationship between undersampled input data and full-sampled output data [21, 24]. Although this simple scheme provides descent results, the reconstruction performance can be improved in various ways. One fundamental approach is the utilization of redundant information contained in MRI data. MRI data are often acquired through multi-channel receiver coils, where the multi-channel data are correlated to each other. Additionally, data acquired in neighboring slices or consecutive time frames are much similar. Moreover, different contrast images contain similar structural information. All the mentioned redundancies can be utilized to boost the reconstruction performance, preserving fine details in the images. Therefore, it is worthwhile to outline recent developments in the field of accelerated MRI reconstruction, especially for cases exploiting the redundant information.

This paper is structured as follows: Sect. 2 outlines the fundamentals of deep learning techniques, while Sect. 3 explains the basics of accelerated MRI including the mathematical model, conventional reconstruction methods, and deep learning-based reconstruction methods in a general sense. Section 4 describes the deep learning-based reconstruction methods utilizing redundant information and finally, Sect. 5 discusses the challenges and limitations followed by the conclusion.

## Basics of deep learning

As a subset of machine learning, deep learning [25] mimics the human nervous system by utilizing a multilayer neural network with numerous processing units, attempting to extract representations of the input data. The difference between conventional machine learning and deep learning lies in the data feature extraction methods: while conventional machine learning relies on annotations by experts, deep learning depends on the network for feature extraction [4]. Both approaches require extraction of meaningful representations by the neural network to achieve accurate results [26]. For training the network, several learning strategies have been developed, motivated by human learning patterns.

### Types of learning

In general, machine learning and deep learning can be categorized into five types according to learning policy: supervised, unsupervised, semi-supervised, self-supervised, and reinforcement learning. Supervised learning utilizes labeled data for network training. The network learns to directly map the input data to the target data. With a large amount of well-labeled data, supervised learning approaches have shown outstanding performances in medical fields [27–30]. However, labeled data are not always available. In such cases, unsupervised learning is often employed, where the network is trained with unpaired data [31–33]. The network tries to learn the correlation between the input and output data distributions. In cases of scarce-label setting, semi-supervised learning techniques are used [34, 35]. Unpaired data are utilized along with a small amount of labeled data, guiding the network to learn the target data distribution. Recently, self-supervised learning has been laboriously researched in situations where labels are not obtainable. The technique aims to utilize the key information of the input data for the generation of missing or required signals. Self-supervised learning approaches [36, 37] have shown superb performance despite the limited amount of data. On the other hand, reinforcement learning [38] updates the network parameters depending on actions that result in rewards or penalties. The network is trained to achieve the optimal result.

### Network layers

Current deep neural networks contain a substantial number of layers to yield high performance, owing to advances in computing power and database. The networks use combinations of layers to extract meaningful features from the input data. One widely used layer is the convolutional layer [39], which extracts features by performing convolution operation with multiple convolution kernels. Each kernel shares the same weights for different parts of the input, motivated from the fact that an image contains similar spatial structures. Accordingly, the convolutional layer utilizes local information for the feature extraction. The pooling layer acts as a down-sampling layer, thus reducing the computational cost of the network. A max-pooling layer or an average pooling layer, which respectively extract the maximum or average values within a given pooling size is employed in most cases. Another one is the activation layer [40], which enables the network to approximate nonlinear functions for describing the relationship between the input and the output. There exist numerous activation functions, in which popular ones include sigmoid, rectified linear units (ReLU), leaky ReLU, and hyperbolic tangent (tanh). On the other hand, the normalization layer alleviates the high sensitivity on hyperparameters and weight initialization of the network during the training process. Normalization layers such as batch normalization [41], layer normalization, instance normalization and group normalization [42] are also important component of the deep neural network. In addition, the fully-connected layer utilizes all the neurons from the previous layer. The layer integrates all the local information, often used as a classifier [43].

### Deep learning models

As networks get wider and deeper, training and optimization of the networks become more difficult due to a number of issues including vanishing gradient [44], overfitting [45], and memory constraints. To overcome these problems, deep neural networks have steadily evolved in various research fields [46–48]. In this section, a few widely implemented deep learning models are introduced.

**Fig. 1:**
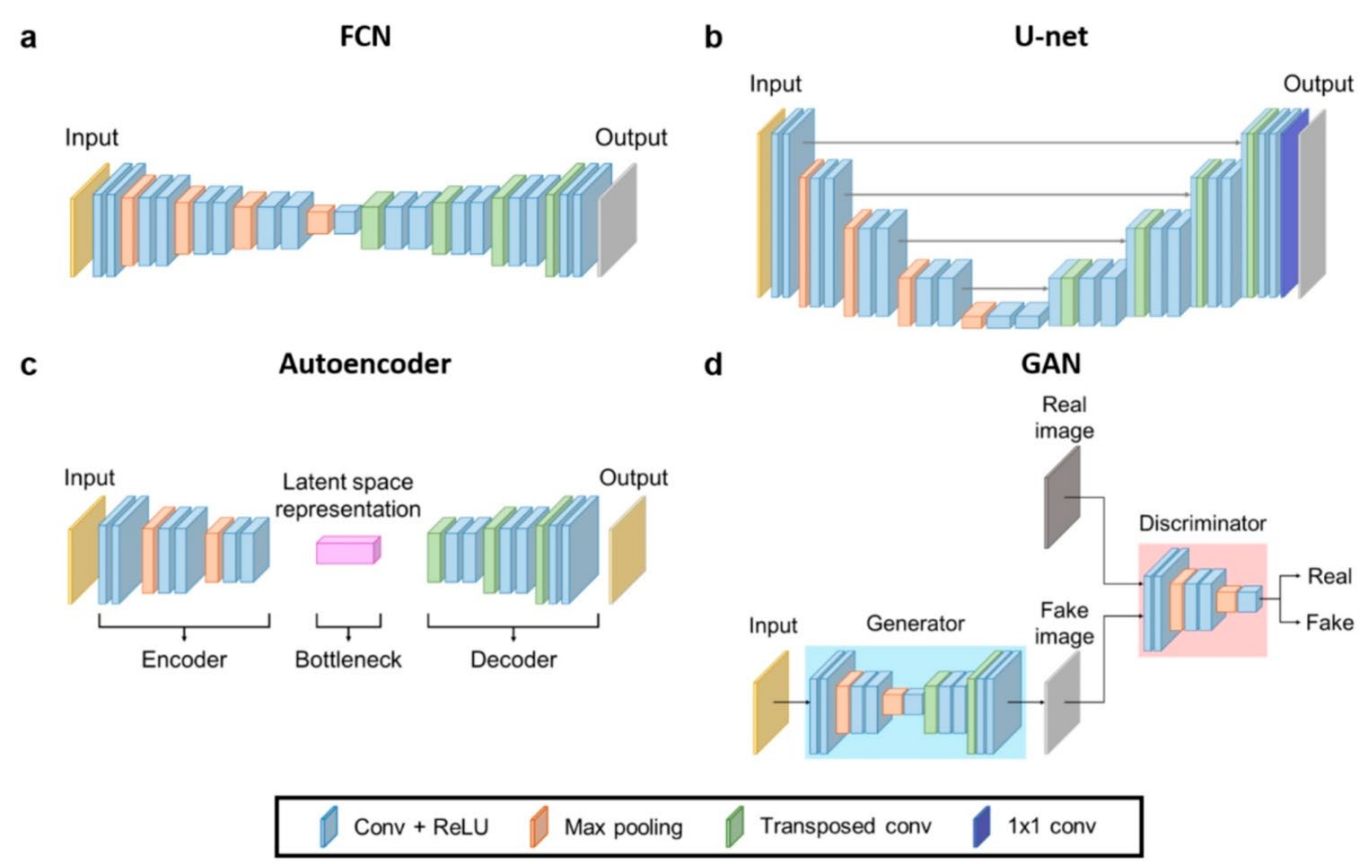
Illustrations of various neural networks: **a** fully convolutional network (FCN), **b** U-net, **c** autoencoder, and **d** generative adversarial network (GAN). The FCN and U-net aims to learn meaningful features to produce target output, whereas the autoencoder aims to learn meaningful latent space representation, trained to produce output that is identical to the input. The generator of GAN is trained to produce realistic output while the discriminator is trained to discriminate between generated data and the reference data

Combinations of different layers and connections result in diverse network architectures and appropriate models were proposed for specific tasks. A convolutional neural network (CNN) has been dominantly employed in medical image analysis for years, due to the large spatial dependences between medical data. Accordingly, 2D-CNN [49] and 3D-CNN [50, 51] models are used in many applications including classification and segmentation. Despite the memory and time efficiency of 2D-CNN models, 3D-CNN models yield higher performance by utilizing more information from the 3D input data. Variants of CNN architectures include the fully convolutional network (FCN) [52] and U-net [8], which are two fundamental models used for image segmentation (Fig. 1a, b). Both models have an encoder-decoder structure, where meaningful features are extracted through the encoder and a segmentation map or image is restored in the decoder part. Additional residual paths are introduced between the encoder and the decoder in U-net for better utilization of the feature maps and faster convergence. Both models are broadly used in medical applications [53–56].

Autoencoder (AE) [57] consists of two networks, where the first part encodes the input into the latent space and the other restores the input (Fig. 1c). The objective is to produce an output that is identical to the input. Through training, the encoder part learns to map the data into the latent space with meaningful information of the input data. Consequently, the AEs are utilized for dimensionality reduction and feature extraction. There exist many variants of AE including denoising AEs [58] and stacked AEs [59], which are employed in various tasks [60–62].

Generative adversarial networks (GANs) [63] are also widely used deep learning models (Fig. 1d). The generator is trained to produce a realistic image, whereas the discriminator is trained to distinguish if the input image is real or fake. GAN tries to learn the underlying data distribution through min-max optimization, by training the generator and discriminator in an adversarial manner. Despite their training instability, GAN models are widely applied across various research fields, and numerous modified architectures [64–66] have been developed. This is also true in medical fields, where GAN models are implemented in tasks such as image synthesis [67] and denoising [68].

**Fig. 2:**
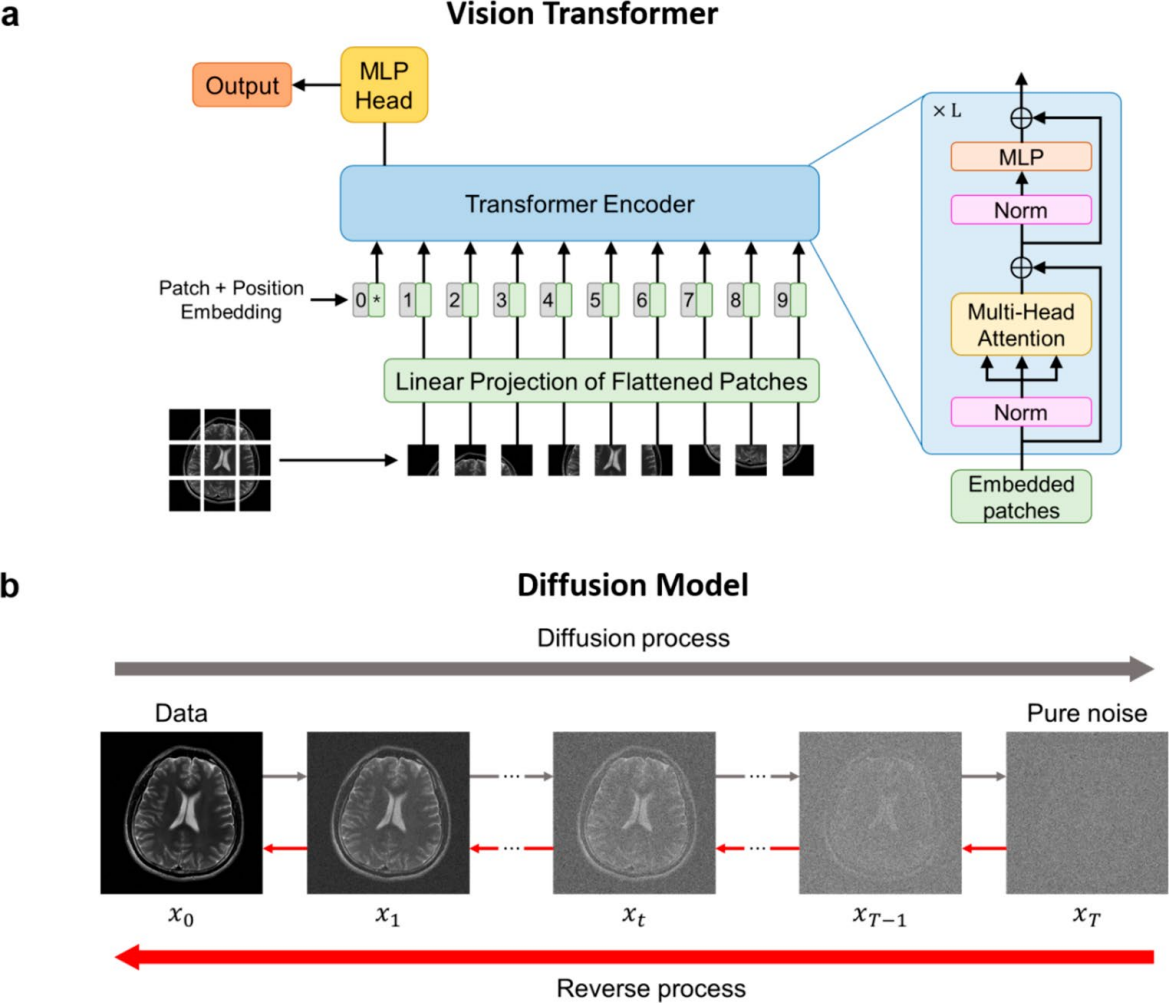
Recent advances in deep neural networks. **a** Illustrations of vision transformer network. The image patches are linearly embedded and fed into the transformer encoder with position embedding. The encoder is repeated L times and the produced output goes through the MLP head, resulting in the class output. Norm stands for normalization and MLP stands for multilayer perceptron. **b** Illustration of diffusion model. The image goes through the diffusion process, where noise is added at each time step until the data becomes pure noise. The reverse process removes the noise at each time step to obtain desired output image

Recently, the transformer [69] has been developed and demonstrated superior performance compared to CNNs. The transformer is composed of an encoder and a decoder, which contain multiple layers of self-attention and feedforward networks. The transformer was initially developed for natural language processing [70, 71], since the main advantage is the ability to capture the long-range dependencies of the input sequence. Consequently, transformers rapidly replaced CNNs due to the inherent limitations of the latter including limited effective receptive fields. In image processing, vision transformer [72] was introduced, which divides the input image into patches of a fixed size (Fig. 2a). These patches are treated as an input sequence with additional patch embedding. Vision transformers have shown superior performances not only in many visual tasks [73–76], but also in medical imaging areas [77–80].

Diffusion probabilistic models (DPM) [81, 82] have recently attracted huge attention as a new family of generative models. DPMs are utilized not only in computer vision [83–85] but also in the medical imaging field [86–88], due to their well-established mathematical foundations. Moreover, they allow stable training and controllable generation, which makes DPMs more desirable than GANs [89]. DPM contains two main processes: the diffusion process and the reverse process (Fig. 2b). The diffusion process perturbs the data with normally distributed noise in each time step until it is transformed into pure noise, whereas the reverse process recovers the clean data by estimating and removing small noise perturbations in each time step. The models are further divided into categories based on the variational perspective and the score-based perspective. The variational perspective typically minimizes the Kullback-Leibler divergence between predicted and target distributions, while the score-based perspective utilizes the score function of the log-likelihood of the data in the diffusion process [90]. DPMs have shown outstanding performance in MRI reconstruction, since the reverse process allows denoising and reconstruction simultaneously.

## Accelerated MRI

Accelerated MRI techniques have been implemented in clinical practices for a long time, reducing the scan time. To understand how accelerated MRI has played such an essential role in clinical applications, it is important to know the basics and brief history. Therefore, we first start with the mathematical model for MR image reconstruction, and then cover conventional and general deep learning-based reconstruction methods in this section.

### General model

The acquired MRI signal can be modeled as follows:

1 $$\:y=Ax+\epsilon,$$

where $\:y$ is the acquired undersampled k-space data, $\:x$ is the image to be reconstructed, $\:A$ is the forward operator of MRI, and $\:\epsilon$ is the additive noise. In the single coil case, $\:A={\Omega\:}\mathcal{F}$, where $\:{\Omega\:}$ represents the undersampling operation in k-space and $\:\mathcal{F}$ indicates the Fourier transform. In the multi-coil setting, $\:A={\Omega\:}\mathcal{F}C$, where $\:C$ is the coil sensitivity map.

When full-sampled k-space data are available, Eq. (1) can be solved by directly applying the inverse Fourier transform. However, the problem becomes ill-posed when k-space is undersampled, not satisfying the Nyquist sampling theorem. In this case, the reconstruction problem can be formulated as an optimization problem with an additional constraint.

2 $$\:{x}^{*}=\underset{x}{\text{argmin}}\frac{1}{2}{\parallel Ax-y\parallel}_{2}^{2}+\lambda\:\mathcal{R}\left(x\right),$$

where $\:\lambda\:$ is the regularization parameter and $\:\mathcal{R}\left(x\right)$ is the regularization term. There are several choices for $\:\mathcal{R}\left(x\right)$, including wavelet transform, total variation, and low-rank structures [91–94]. For deep learning-based reconstruction, the regularization can be achieved as a property of the neural network itself. On the other hand, the first least-squares term is the data consistency term, enforcing the consistency with the acquired data.

### Conventional reconstruction methods

**Fig. 3:**
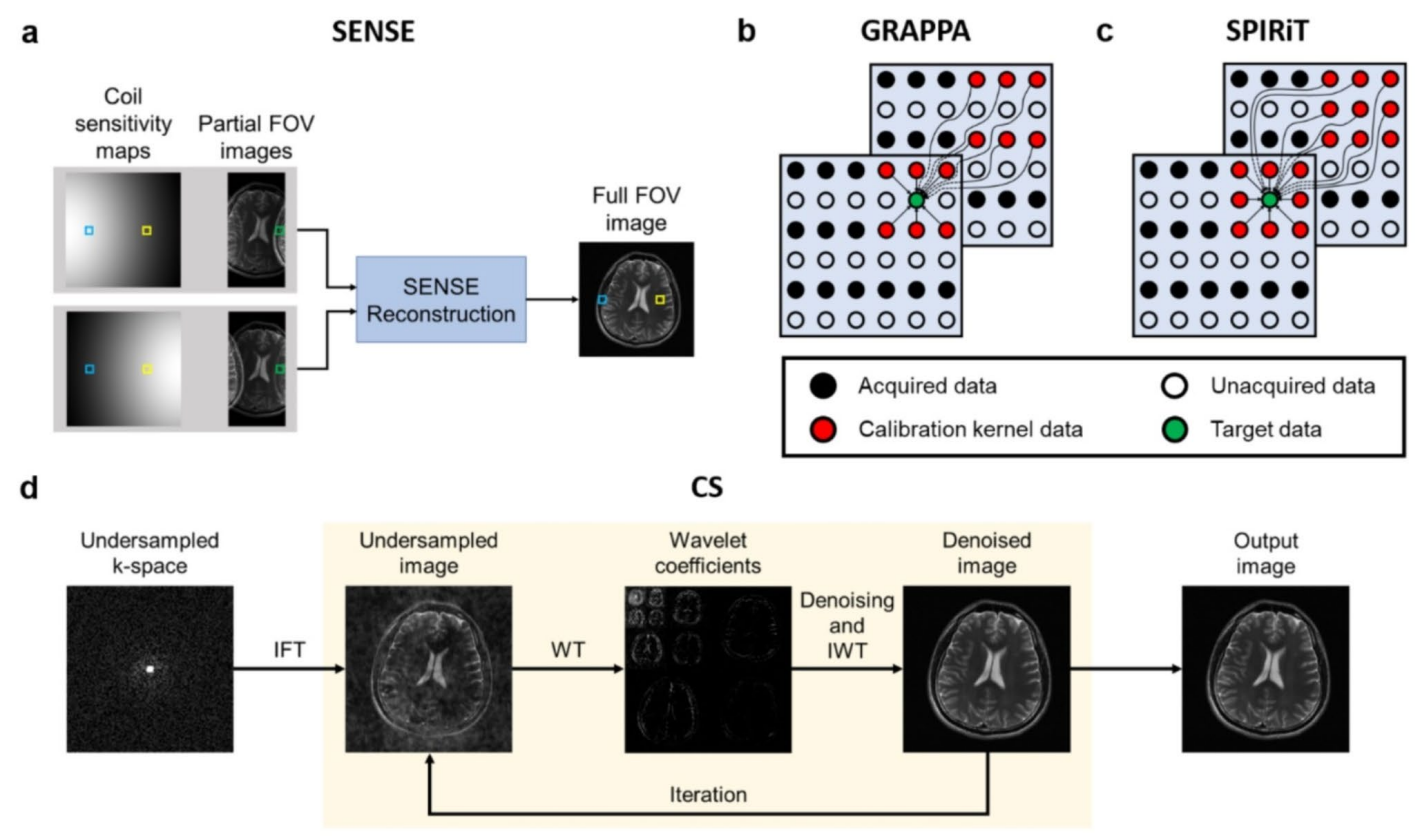
Conventional accelerated MRI reconstruction methods. **a** sensitivity encoding (SENSE) method, in which coil sensitivity maps and partial field-of-view (FOV) images are utilized to resolve aliasing artifacts in image domain. Data at blue and yellow boxes of the full FOV images are estimated using corresponding data in coil sensitivity maps and the data at green boxes in the partial FOV images. **b** calibration kernel used in generalized autocalibrating partially parallel acquisitions (GRAPPA) method. Only the acquired data points are used for the estimation of missing data. **c** calibration kernel used in iterative self-consistent parallel imaging reconstruction (SPIRiT) method. All the neighboring data points are used for the estimation of missing data. **d** compressed sensing (CS) method, where an undersampled image is transformed into wavelet domain and denoised iteratively to reconstruct a full-sampled image. IFT indicates inverse Fourier transform, WT indicates wavelet transform, and IWT indicates inverse wavelet transform

PI is widely used in clinical applications, exploiting the spatial sensitivity maps of multi-coil data to reconstruct images. Numerous PI techniques have been developed: some operate in the image domain to resolve the aliasing artifacts, while others work in the k-space domain to estimate the missing k-space signals. Sensitivity encoding (SENSE) [1] is a reconstruction method that processes in the image domain by utilizing the coil sensitivity map (Fig. 3a). On the other hand, GeneRalized Auto-calibrating Partially Parallel Acquisitions (GRAPPA) [2] estimates the missing data in k-space (Fig. 3b). The method fully samples central k-space lines, namely the auto-calibration signal (ACS). These ACS data are used to estimate the GRAPPA kernel weights, in which the kernel is used to fill the missing data in k-space. The missing data are locally estimated using the acquired data and the GRAPPA kernel in an iterative manner. SPIRiT [95] is a more generalized technique of GRAPPA, which utilizes the weighted sum of neighboring points from any sampling trajectory (Fig. 3c).

CS [3] is also a broadly accepted reconstruction approach, which relies on sparsity representation in another transform domain and related incoherent k-space sampling (Fig. 3d). Since sampling of the k-space center portion is important for CS performance, Cartesian sampling patterns often have higher weighting on the k-space center portion than the edge portions, and radial or spiral trajectories are used frequently for CS [96, 97]. In general, CS works better with 3D or 4D imaging than 2D imaging because of spatiotemporal redundancy. For 3D and 4D imaging, diverse sampling patterns are available and the incoherence can be more optimized [98]. A lot of efforts have been put into application of CS in dynamic MRI [99–101]. Moreover, CS and PI could be combined [95, 100, 102, 103], and further advances considering golden-angle radial sampling [104] or annihilating filter-based low-rank Hankel matrix (ALOHA) [105] were suggested.

The dictionary learning-based method proposed in [106] utilizes sparse representations as well for reconstruction. The approach iteratively solves the optimization problem with a limited amount of data. Each iteration step includes updating the dictionary and sparse representations, followed by updating the image reconstruction. The K-SVD algorithm [107] was employed for the learning scheme, showing superior reconstruction quality compared to CS-based methods using nonadaptive wavelets and total variation. More recent works includes [108], where simultaneous reconstruction of multi-contrast images was achieved by the proposed coupled dictionary learning.

Despite the fact that these conventional approaches are widely used in clinical practices, they have many limitations. For PI techniques, the main drawback is the noise amplification caused by the reconstruction of high frequency information from undersampled data [109]. Additionally, several artifacts may occur, which includes ghosting artifact and geometric distortion artifact [109]. On the other hand, CS approaches introduce global ringing artifacts and image blurring, which degrade the quality of the reconstructed images [110]. Other challenges include achieving incoherence and the long reconstruction time required to solve the nonlinear optimization problem [111]. The dictionary learning-based methods also require a lengthy iterative process, without guarantee of convergence [112]. Due to these challenges, the acceleration rate of the above techniques in clinical applications is typically limited to a range between 2 and 6.

### General deep learning-based reconstruction methods

Deep learning was adopted quickly for MR image reconstruction. In the early stages, residual networks and U-nets were implemented but soon after that, more specialized approaches and network architectures have been proposed. Generally, deep learning-based MRI reconstruction methods can be divided into two categories. The first approach is data-driven end-to-end learning, which aims to learn the relationship between undersampled data and full-sampled data. The other is the model-driven unrolling iterative deep learning method, in which traditional iterative algorithms are utilized to solve the inverse problem.

#### Data-driven end-to-end learning

Data-driven end-to-end deep learning methods aim to learn the mapping from undersampled data to full-sampled data with a large amount of training data. The approach can further be categorized into reconstructions in image domain and k-space domain. In general cases, reconstruction in image domain provides better robustness to noise and artifacts. De-aliasing GAN (DAGAN) [113] is an example of reconstruction in image domain using U-net based generator, in which the authors employ additional content loss to preserve details of the images. Other examples include enhanced recursive residual network (ERRN) [114] that employs ResNet [115] for MR image reconstruction, DeepcomplexMRI [116] that utilizes relationship between real and imaginary values of the complex images, and RefineGAN [117] that implements deeper networks with cycle consistency loss for accurate reconstruction.

**Fig. 4:**
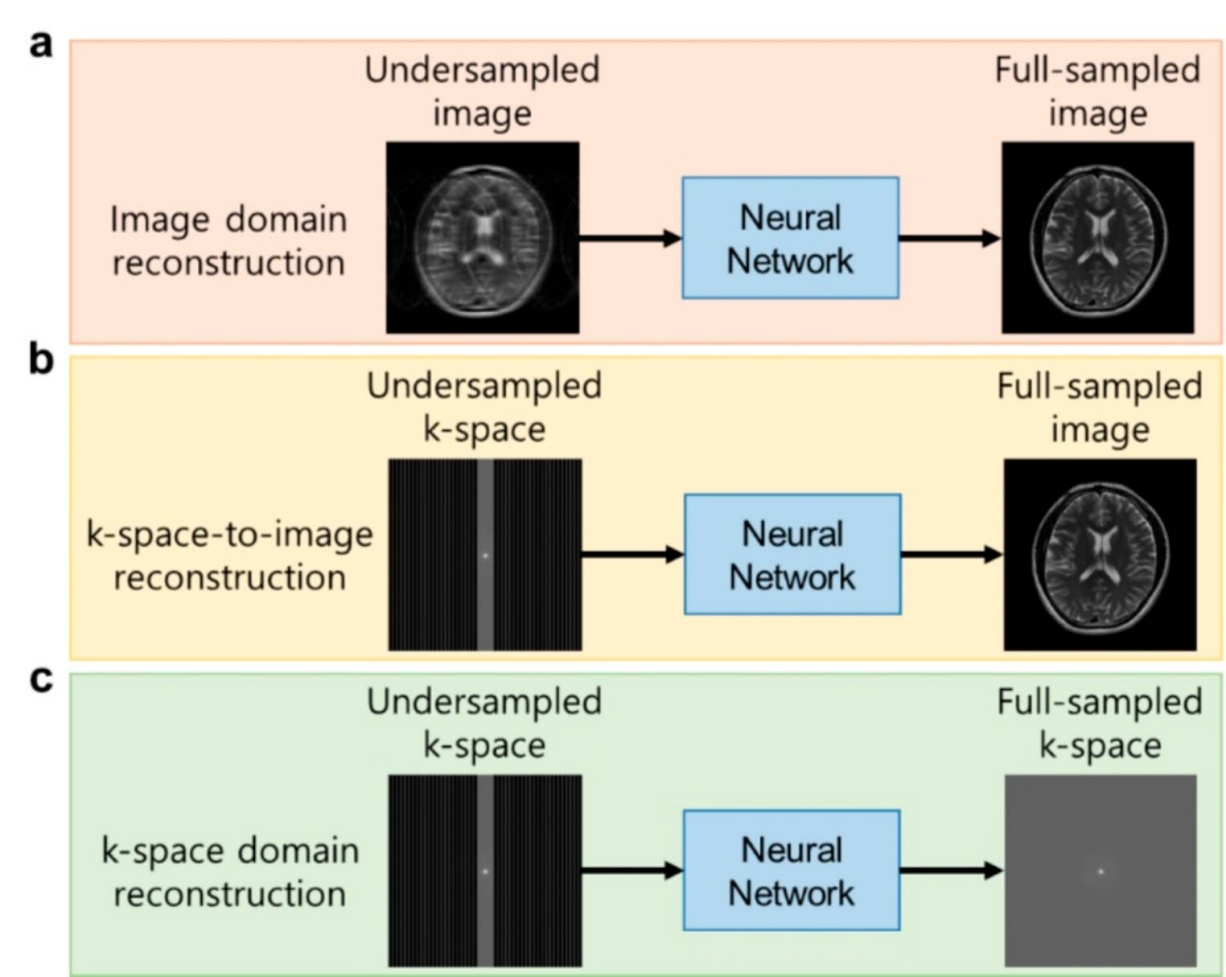
Various approaches for data-driven end-to-end learning for accelerated MRI reconstruction. **a** image domain reconstruction, where both the input and the output are in image domain. **b** k-space-to-image reconstruction, where the input is in k-space domain and the output is in image domain. **c** k-space domain reconstruction, where both the input and the output are in k-space domain

On the other hand, reconstruction in k-space better preserves high-frequency information. Robust artificial-neural-networks for k-space interpolation (RAKI) [118] is one example, which utilizes a convolutional network that works as a GRAPPA kernel to interpolate the missing k-space lines using ACS lines. Another approach [119] was developed based on RAKI, implementing a cyclic architecture and utilizing information from neighboring slices to provide noise-robust reconstruction with a small number of ACS lines. Since reconstructions in image domain and k-space domain have distinct advantages, various methods utilizing information from both domains were also suggested [120–123]. Various end-to-end learning schemes are illustrated in Fig. 4.

#### Model-based unrolled iterative deep learning method

**Fig. 5:**
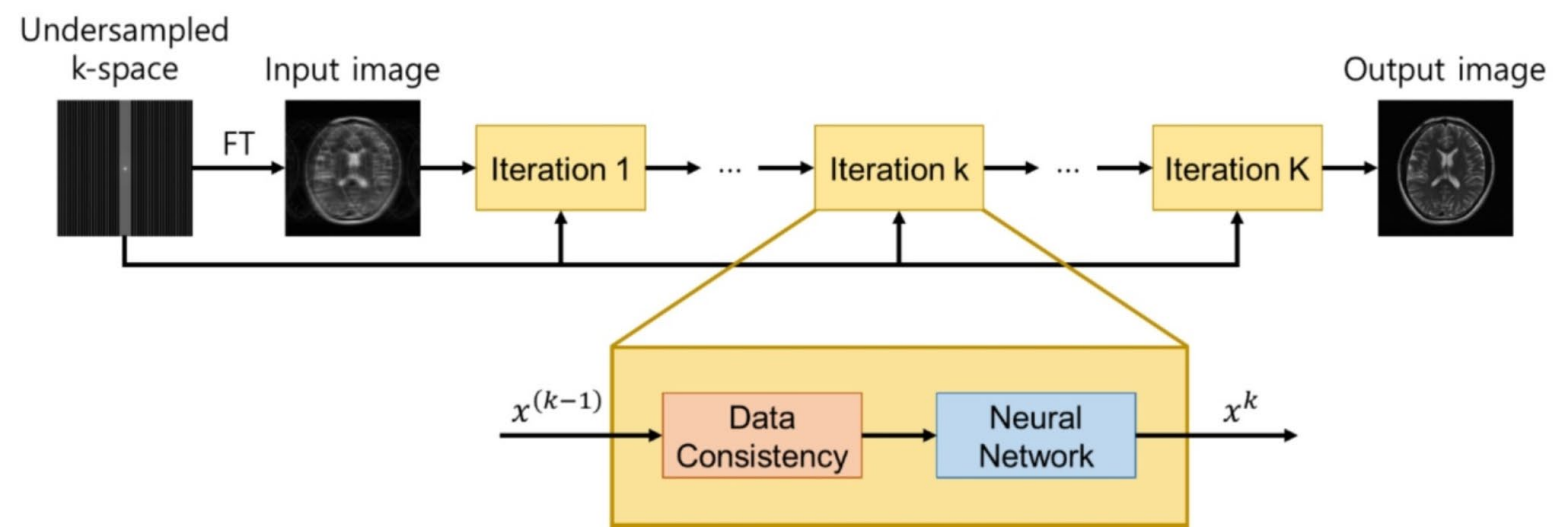
Diagram of model-based unrolled iterative deep learning method. The scheme involves iterations of the data consistency block and the regularization block, in which the neural network is implemented for regularization

Along with data-driven end-to-end learning, the model-based unrolled iterative deep learning is another popular approach for MR image reconstruction. The approach consists of three parts: the physics-based model, the optimization algorithm, and the deep neural network. The process starts by formulating the mathematical model based on MR physics, followed by setting up an appropriate optimization algorithm for reconstruction. Lastly, the network is trained to learn the functions in the model. The scheme for model-based unrolled iterative deep learning method is illustrated in Fig. 5.

A wide variety of model-driven methods have been proposed using different optimization algorithms. One of the first works utilized alternating direction method of multipliers (ADMM) algorithm for CS-MRI [124]. The ADMM-Net successfully learned the regularization term via ADMM algorithm and showed high reconstruction performance. Based on this work, other variants using ADMM algorithm were proposed [125, 126]. Other optimization algorithms include primal dual hybrid gradient (PDHG) algorithm [127] and iterative shrinkage-thresholding algorithm (ISTA) [128]. In [129], inspired by dictionary learning reconstruction, cascade of CNNs with distinct weights were implemented as regularization terms. The model outperformed conventional dictionary learning-based method by utilizing separate CNNs in each iteration. In another work, the model-based reconstruction using deep learning prior (MoDL) [130] was proposed, which used denoiser as learning objective. MoDL captured redundant information in the image, showing superb performances in various works [36, 131, 132].

The gradient descent algorithm was implemented in many works. One is the variational network [133]that implemented the Fields of Experts model, a generalization of total variation, as the regularization term. The regularization parameters including the filter kernels, activation functions and the weight for data consistency term were all learned from the data. End-to-end variational network (E2E-VarNet) [134] was then developed, where the U-net was used rather than the gradient of the Fields of Experts model. Furthermore, the coil sensitivity maps were estimated through another U-net. This boosted the reconstruction performance by accurately predicting sensitivity maps with small amount of ACS lines, which was not achievable in the original work. On the other hand, Hosseini *et al*. [135] implemented dense recurrent neural networks using proximal gradient descent unrolling scheme to facilitate the information flow. Consequently, the proposed model achieved higher reconstruction quality without extra computational power.

## Deep learning-based reconstruction methods utilizing redundant information

As mentioned previously, clinical images including MR images have large spatial correlations. The spatial correlation is essential for accelerated MRI with its sparse sampling, for the reconstruction of high-quality images. The reconstruction performance can further be improved by utilizing the redundant information or correlation across various domains. Multi-coil MR imaging allows to utilize the redundant information across signals acquired by multiple receiver coils, which are generally employed with multi-channel inputs in deep learning applications. Since appropriate utilization of MR image redundancy may significantly improve MRI acceleration, we discuss about the deep learning methods that exploit redundancy across multi-contrast MRI and the redundancy across spatial and temporal domains in this section.

### Redundancy across multiple contrast images

Different MR contrast images introduce different information demanded in various clinical diagnosis. For instance, T1-weighted images depict anatomical structures very clearly, whereas T2-weighted images focus on pathology and are used to detect abnormalities. Although different contrasts convey different diagnostic information, they contain similar structural information. Consequently, multi-contrast images are utilized in various researches. As an example of multi-contrast image synthesis, multi-task deep learning (MTDL) model synthesizes six contrasts from eight echoes of a multi-dynamic multi-echoes (MDME) sequence [136]. On the other hand, an efficient diffusion model for multi-contrast MRI super-resolution (DiffMSR) employs both diffusion model and transformer block for MR image super-resolution [137]. This redundant information can also boost the accelerated MRI reconstruction performance. Accordingly, this subsection reviews the literature on MRI reconstruction exploiting multi-contrast redundancy (Table 1). More specifically, the subsection is further divided into two categories: works that use full-sampled data as guidance and those that use undersampled data only.

**Table 1:** Summary of the reviewed deep learning-based methods utilizing redundancy of multi-contrast MRI data. CNN indicates convolutional neural network and GAN indicates generative adversarial network. Unrolled indicates model-based unrolled iterative scheme, where inside the parenthesis indicates the backbone network.

Reference	Network	Reconstruction domain	Full-sampled input data	Acceleration rate (*R*)	Undersampling pattern
Do *et al*. [148]	CNN	Image	Depends on the model	$\:\le\:8$	1D Cartesian random, uniform, and central undersampling
Lee *et al*. [149]	CNN	Image	Depends on the model	2, 4	1D Cartesian uniform undersampling
Pooja *et al*. [155]	CNN	k-space-to-image	X	4	1D Cartesian random undersampling
Xiang *et al*. [138]	U-net	Image	O	8	1D Cartesian Gaussian, uniform, and central undersampling
Falvo *et al*. [139]	U-net	Image	O	4	1D Cartesian undersampling (80% central, 20% uniform)
Yang *et al*. [140]	U-net	Image	O	4, 8	Optimized undersampling pattern by proposed method
Seo *et al*. [156]	U-net	Image	X	8, 10.67	Optimized undersampling pattern by proposed method
Liu *et al*. [141]	Unrolled (CNN)	Image	O	8, 16	1D Cartesian Gaussian undersampling
Liu *et al*. [142]	Unrolled (CNN)	Image	O	8, 16	1D Cartesian random undersampling
Sun *et al*. [152]	Unrolled (CNN)	Image	X	5, 10	1D Cartesian undersampling for *R* = 5, 2D random undersampling for *R* = 10
Guo *et al*. [154]	Unrolled (CNN)	Image	X	$\:4\le\:\text{R}\le\:6.67$ for 1D sampling, $\:5\le\:\text{R}\le\:10$ for 2D sampling	1D Cartesian, 2D random undersampling
Lei *et al*. [143]	Variational network	Image	O	4	1D Cartesian random undersampling
Polak *et al*. [153]	Variational network	Image	X	6 for 2D data, $\:4\times\:4$ for 3D data	Complementary k-space undersampling (contrast-dependent uniform undersampling)
Kim *et al*. [144]	GAN	Image	Depends on the model	4, 8	1D Cartesian central undersampling
Dar *et al*. [145]	GAN	Image	O	$\:\le\:50$	Variable-density undersampling [111]
Wei *et al*. [146]	GAN	k-space-to-image	O	4, 8	1D Cartesian random undersampling (mostly central lines)
Kim *et al*. [151]	GAN	Image	X	4, 8	1D Cartesian central and random undersampling
Zhou *et al*. [80]	Transformer	k-space-to-image	O	2, 4, 6, 8	1D Cartesian random undersampling
Li *et al*. [147]	CNN + Transformer block	Image	O	8, 16	1D Cartesian random and uniform undersampling

#### Full-sampled data guidance

In the early stages, U-net based architectures were implemented in many works. Xiang *et al*. [138] implemented Dense U-net to accelerate the acquisition of T2-weighted images using full-sampled T1-weighted images as guidance. This work simply concatenated multi-contrast images for the network input but in [139], the authors fed undersampled T2-weighted image and full-sampled FLAIR image to the Dense U-net as separate inputs. The features were extracted separately and merged in the mid-stage of the encoder to produce full-sampled T2-weighted image with higher quality. Yang *et al*. [140] proposed three-stage network that optimized the sampling pattern of target contrast and reconstructed the image at once using U-net based networks. The first stage synthesized the target contrast image using three contiguous slices of the reference contrast and calculated the residual in k-space domain. The k-space residual was used in the second stage along with learnable parameters to determine the optimal sampling pattern. The reconstruction was proceeded in the last stage, which produced high quality image. The model exploited the difference between the produced image and the label in k-space, providing robustness to the misregistration of different contrast images.

Model-based or physics-based unrolled iterative methods show excellent robustness, thus actively utilized for multi-contrast reconstruction. In [141], full-sampled T2-weighted image was reconstructed from full-sampled T1-weighted image and undersampled T2-weighted image with an iterative scheme. By using the feature extraction block and dilated inception block in the regularization unit, the proposed method achieved higher performance than the comparison methods for acceleration rates of 8 and 16. Meanwhile, a spatial transformation module and normalized cross correlation were utilized in [142] for reconstruction. Using an unregistered reference image removed the need for separate registration of input images. In [143], the multi-contrast variational network (MC-VarNet) was proposed using the half-quadratic splitting algorithm, performing reconstruction and super-resolution simultaneously. MC-VarNet has shown higher performance and robustness against noise and irregular reference images than other deep learning-based methods.

Multi-contrast MRI reconstruction was also thoroughly researched by utilizing generative models. GAN-based reconstruction method was developed in [144], which used pixel-wise loss and adversarial loss. It produced images that were perceptually better than those reconstructed by the previous methods. Another approach utilized low frequency components of the target contrast image and high frequency components of another contrast image for reconstruction [145]. The proposed model successfully recovered pathologies that were unclear in the input image and enabled high acceleration rate up to 50. In [146], the double-domain synthetic network was proposed, exploiting both image and k-space domains. The model was composed of two stages based on GAN framework. The first stage generated full-sampled k-space, which was used for the discriminator trained in k-space domain and for the second stage after inverse Fourier transform. The second stage enhanced the image from the first stage through another generator, followed by separate discriminator trained in the image domain. The double-domain network effectively reduced aliasing artifacts while recovering detailed structures for acceleration of multi-contrast MRI.

For further improvements, transformers were implemented for multi-contrast MRI reconstruction. In [80], dual-domain self-supervised transformer (DSFormer) was proposed. The network was trained in a self-supervised fashion, in which the undersampled k-space was divided into two partitions in the initial stage. The reconstruction was processed in the image domain but the loss was given in the k-space domain, utilizing both domains for reconstruction. The authors implemented a cascaded Swin transformer reconstruction network with a data consistency block for high-fidelity reconstruction. On the other hand, multi-contrast complementary information aggregation (MCCA) network [147] performed multi-scale feature fusion along with hybrid convolutional transformer module to improve performance and generalization ability of the network.

#### Without full-sampled data guidance

**Fig. 6:**
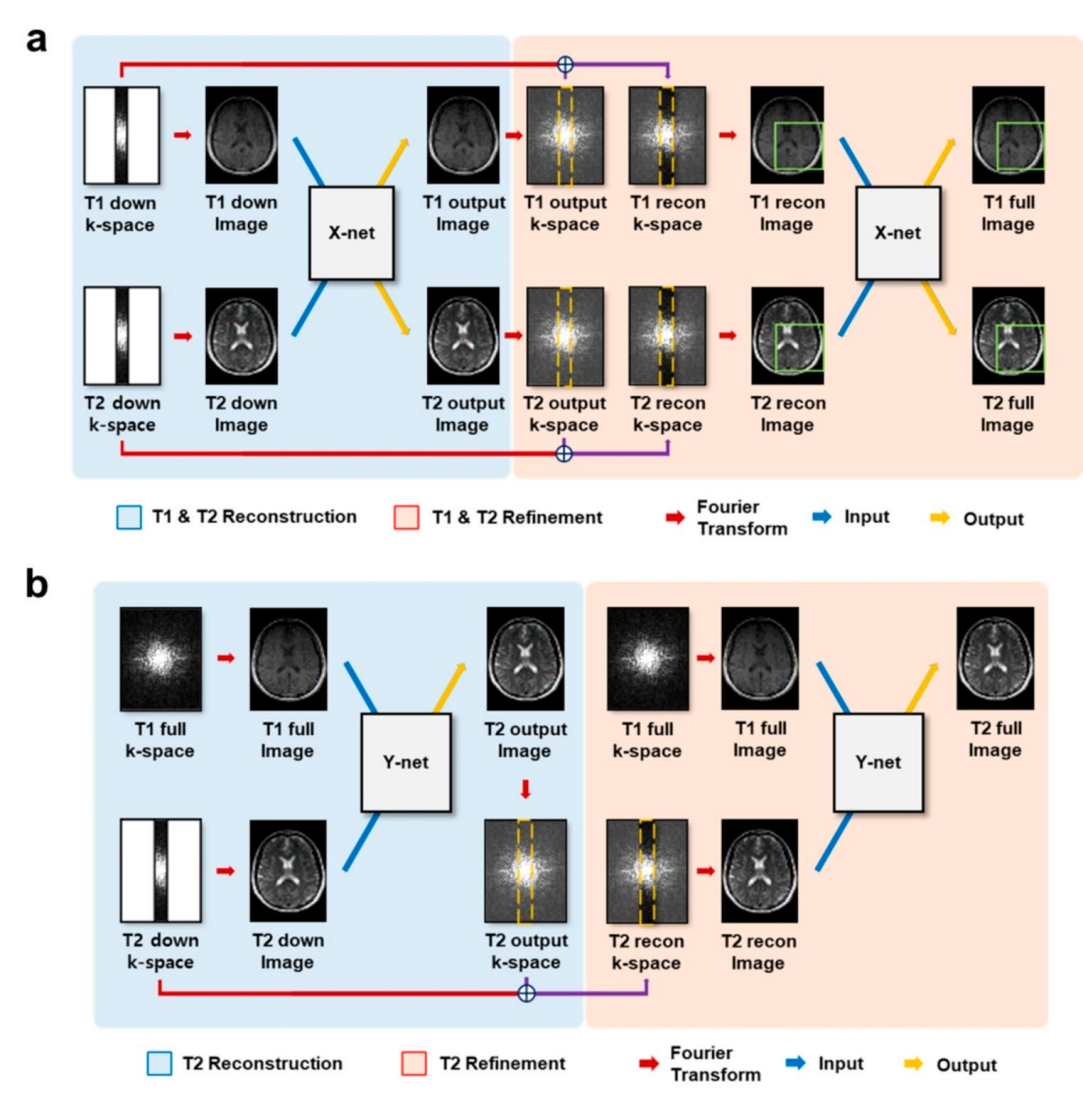
Illustrations of X-net and Y-net from Do *et al*. [148]. **a** X-net framework. Undersampled T1-weighted image and T2-weighted image are reconstructed with the first X-net and the k-space data of each output from the first X-net is combined with the input k-space data for data consistency. This is followed by another X-net trained with patch augmentation for refinement. **b** Y-net framework. Full-sampled T1-weighted image and undersampled T2-weighted image are used in the first Y-net to reconstruct full-sampled T2-weighted image. The k-space data of the output from the first Y-net is combined with the input k-space data and used to train the second Y-net for refinement

Despite the fact that utilizing full-sampled data as a guidance yields higher reconstruction performance, approaches using only undersampled data have been proposed for settings where full-sampled data are not available. Do *et al*. [148] proposed two frameworks, namely the X-net without guidance of full-sampled data and the Y-net with the guidance (Fig. 6). The networks were cascaded two times for complete reconstruction and showed higher performance than the U-net. Later, the networks were utilized for metal artifact correction [149], demonstrating higher performance than the conventional CNN approach [150] by exploiting multi-contrast redundancy. For the work using generative model, Kim *et al*. [151] demonstrated improvement in multi-contrast reconstruction by setting the undersampling phase-encoding directions orthogonal across multi-contrast acquisitions.

Model-based unrolled iterative schemes were also widely used for reconstruction without full-sampled data guidance. Deep information sharing network (DISN) [152] was proposed to reconstruct images from three undersampled contrast images: proton density, T1-weighted and T2-weighted images. The model included iterations of the feature sharing block and data fidelity block, in which the large model capacity provided robustness to misregistration. Joint variational network (jVN) [153] was proposed using gradient descent optimization, jointly reconstructing T1-weighted, T2-weighted, and FLAIR images. The authors showed that complementary sampling across different contrasts further boosted the performance, as well as enabling acceleration in both 2D and 3D MRI data. Another research employed the projected fast iterative soft-thresholding algorithm (pFISTA), namely the joint group sparsity-based network (JGSN) [154]. JGSN reconstructed two different contrast images from undersampled inputs by implementing the joint group sparsity constraint module in the learnable sparse transform module. Meanwhile, multi-contrast primal-dual neural network (MC-PDNet) was proposed for reconstruction of T2-weighted, GRE, and FLAIR images [155]. The network received undersampled inputs in k-space domain, processed it in both k-space and image domains, and produced output in the image domain. The authors demonstrated how MC-PDNet achieved higher performance than the models utilizing single contrast and DISN [152]. Furthermore, Seo *et al*. [156] proposed simultaneous sampling pattern optimization and reconstruction of arbitrary number of undersampled contrast images using a joint-MoDL scheme. The sampling patterns were optimized for each contrast and this resulted in less overlapping area than the optimization of single contrast. The authors demonstrated superior reconstruction performance than the other state-of-the-art models, as well as showing the model’s generalizability by applying the brain-trained model to the knee data.

### Spatiotemporal redundancy

Although high dimensional MR imaging requires a longer scan time, the data contains more spatiotemporal redundancy than those acquired by 2D imaging. The neighboring spatial planes show similar structures, and this is also true for the images of consecutive time frames. Taking advantage of the redundant spatial and temporal information may dramatically reduce the scan time for MR imaging of high dimensions. Additionally, the reconstruction performance can be boosted, such as in [119], where noise robustness was improved by exploiting neighboring slices for reconstruction. In spite of the advantages, utilizing the spatiotemporal redundancy in deep learning methods is still a challenge due to the high computational cost. In this subsection, we discuss how recent approaches deal with high dimensional MRI data in regard to the difficulties in implementations and review the relevant literature (Table 2).

**Table 2:** Summary of the reviewed deep learning-based methods utilizing spatiotemporal redundancy. CNN indicates convolutional neural network and GAN indicates generative adversarial network. Unrolled indicates model-based unrolled iterative scheme, where inside the parenthesis indicates the backbone network.

Reference	Network	Data	Acceleration rate (*R*)	Utilized redundancy	Strategy
Wu *et al*. [158]	CNN	3D cartilage data	4, 6, 8	Spatial	Volumetric processing
Küstner *et al*. [176]	CNN	4D body trunk data	≤14	Spatial and temporal	Implementation of 4D convolution as a sequence of 3D spatial and 1D temporal convolutions
Küstner *et al*. [177]	CNN	4D body trunk data	≤14	Spatial and temporal	Implementation of self-supervised learning on [176]
Haji-Valizadeh *et al*. [178]	CNN	4D angiography data	5.5	Temporal	4D data separated into multiple 3D data for network input
Freedman *et al*. [179]	CNN	4D abdominal data	N/A	Temporal	Implementation of 3D convolution to utilize temporal (respiratory phases) information
Kim *et al*. [180]	CNN	4D flow data	$\:\le\:6$	Spatial	Implementation of 3D convolution to utilize spatial information
Man *et al*. [161]	CNN + attention block	Ultralow-field (ULF) 3D brain data	≤2	Spatial	Implementation of 3D convolution and spatial attention block
Du *et al*. [157]	U-net	3D Knee data	4	Spatial	Implementation of slice fusion block to multi-slice input
Le *et al*. [167]	U-net	3D Myocardial perfusion data	≤23	Temporal	Implementation of 3D CNN blocks and residual framework
Murray *et al*. [181]	U-net	4D abdominal data	1.5, 2, 2.25 with respect to XD-GRASP [182]	Temporal	Motion and coil dimension of a slice concatenated in the channel direction for the network input
Yaman *et al*. [159]	Unrolled (CNN)	3D late gadolinium enhancement (LGE) cardiac data	3, 6	Spatial	Divided whole volume into smaller slabs, implementation of 3D convolution
Cho *et al*. [160]	Unrolled (CNN)	3D brain data acquired with various sequences	$\:4\times\:4$, $\:3\times\:3$, $\:4\times\:3$	Spatial	Divided whole volume into smaller slabs, implementation of 3D convolution
Sandino *et al*. [166]	Unrolled (CNN)	Cardiac cine data	$\:10\le\:\text{R}\le\:15$	Temporal	Implementation of 3D convolution as a sequence of 2D spatial and 1D temporal convolution
Miller *et al*. [168]	Unrolled (CNN)	3D ultrashort echo time (UTE) pulmonary data	$\:\le\:10$	Temporal	Implementation of encoder-like architecture to output single time frame from multiple frames before unrolled network
Kellman *et al*. [169]	Unrolled (CNN)	3D knee and brain data	4	Spatial	Hybrid reverse-recalculation and checkpointing scheme for memory-efficient learning
Zhang *et al*. [170]	Unrolled (CNN)	3D koosh ball coronary data	4, 5, 6	Spatial	Distributed learning task on multi-GPUs and implemented mixed-precision processing
Deng *et al*. [171]	Unrolled (CNN)	3D knee data	8, 12	Spatial	Divided whole volume into smaller slabs
Terpstra *et al*. [174]	Unrolled (CNN)	4D lung data	3.7, 7.4, 14.8	Spatial and temporal	Implementation of CNN blocks exploiting spatial and temporal information in parallel
Wang *et al*. [175]	Unrolled (CNN)	4D abdominal data	4, 8, 16, 25	Spatial and temporal	Dictionary learning, extraction of sliding 4D patches by decomposing into two steps of extracting 2D patch in each step
Vishnevskiy *et al*. [172]	Variational network	4D flow data	$\:6\le\:\text{R}\le\:22$	Spatial and temporal	Utilization of 3D filters grouped into four banks to avoid 4D convolutions for regularization term
Qi *et al*. [173]	Variational network	4D Cardiac data	$\:7\le\:\text{R}\le\:10$	Spatial and temporal	Respiratory binning after motion correction
Chung *et al*. [162]	CycleGAN	3D TOF-MRA data	4, 8	Spatial	Two-stage reconstruction: slice-by-slice reconstruction of coronal plane in the first stage, then volume reconstruction of axial plane in the second stage
Chung *et al*. [163]	Diffusion model	3D knee and brain data	2	Spatial	Diffusion-based denoising applied slice-by-slice, while regularization considers z-axis direction only
Lee *et al*. [165]	Diffusion model	3D brain data	8, 24, 48	Spatial	Use of two 2D diffusion models that operate on different planes

#### 3D data reconstruction

Many works dealing with 3D MRI data try to utilize the redundant information in the data volume. In the early stages, simple CNN-based architectures were employed, utilizing only a few neighboring slices due to the memory issues. In [157], multi-coil undersampled k-space knee data of three slices were utilized to reconstruct k-space data of the central slice with U-net-based architecture. The input slices went through the slice fusion block instead of simple concatenation, to better utilize the relation between neighboring slices. The model showed improved performance compared to the GRAPPA and the single slice reconstruction method. In another work, T-net was proposed with additional local residual paths in each stage of encoder and decoder [158]. The approach was applied to neighboring cartilage MRI data with pseudo-random Cartesian sampling and stack-of-stars radial sampling, outperforming traditional compressed sensing approaches in both cases. On the other hand, Yaman *et al*. [159] proposed a self-supervised scheme utilizing sensitivity maps to reconstruct 3D late gadolinium enhancement cardiac MR (LGE CMR) images. This self-supervised approach was especially significant for settings with a small training dataset. Different from the previous works, Cho *et al*. [160] combined wave-encoding and MoDL (wave-MoDL) for acceleration of magnetization-prepared rapid gradient echo (MPRAGE) and multi-echo MPRAGE (MEMPRAGE) acquisitions. Moreover, joint reconstruction of multi-contrast images using 3D-QALAS sequence was demonstrated, showing both rapid acquisition and superb reconstruction performance. Recently, there have been rapid developments for ultralow-field MRI techniques, and several approaches were suggested for acceleration including [161]. The model achieved huge improvements in T1-weighted and T2-weighted image reconstruction by implementing a network with combination of convolutional layers and attention layers. The model preserved fine details in the output images, comparable to the reference images scanned in 3T MRI system.

Several works have implemented generative models to exploit spatial information. Chung *et al*. [162] proposed a two-stage CycleGAN-based model that reconstructed 3D time-of-flight MR angiography (TOF-MRA) data in unsupervised setting. Coronal-view images were reconstructed slice-by-slice in the first stage using MRI physics-driven CycleGAN, then stacked and resized to axial-view volume data, which were refined in the second stage using another CycleGAN-based network. Additionally, a projection discriminator, trained to learn the distributions of volumetric and max-pooled images, was implemented in the second stage to significantly improve the resulting quality of the images. In another work, score-based diffusion model was used to solve for 3D inverse problems [163]. The diffusion model-based iterative reconstruction (DiffusionMBIR) proposed here performed the denoising step in the diffusion model slice-by-slice and the ADMM [164] update step in 3D volume. Consequently, the generative prior was augmented with the model-based sparsity prior, which contributed in producing high quality 3D reconstruction. This work was followed by the two perpendicular 2D diffusion models (TPDM) [165], which achieved to fully learn 3D generative prior. For TPDM, two 2D diffusion models were implemented; the primary 2D diffusion model is trained with sliced images in one plane to solve the inverse problem, while the auxiliary 2D diffusion model is trained for correcting inconsistencies, caused by the primary diffusion model, in another plane. The proposed model outperformed other state-of-the-art 3D reconstruction methods, even with limited amount of data.

There are applications that require temporal information, such as cardiac cine imaging and flow MRI. In such cases, 3D data with time frames as one axis are obtained to observe the true variations in a slice. By using temporal redundancy, the data acquisition can be accelerated as well as improving the reconstruction performance. In [166], temporal information was exploited on cardiac cine dataset. The extended coil sensitivity information using ESPIRiT [201] was utilized to alleviate FOV limitations in the model-based unrolled iterative scheme. Two 3D U-nets were implemented in [167] for the reconstruction of dynamic radial myocardium perfusion images. The model contained residual paths among two networks, providing increased network robustness. The work showed significance in that it can aid in clinical applications with highly time-sensitive environments, such as for patients with coronary artery disease. Another work has shown reconstruction of 3D ultrashort-echo time (UTE) pulmonary data by implementing a model-based deep learning model trained in a self-supervised manner [168]. The model combined several respiratory states for high-quality reconstruction of end-inspiratory phase images. Through this approach, an image of a user-selected respiratory phase could be reconstructed from free breathing acquisitions.

Due to the difficulties in handling high-dimensional MRI data, many works have focused on developing memory-efficient approaches. A memory-efficient learning procedure was proposed in [169], applied to the model-based unrolled iterative framework. The proposed procedure suggested a hybrid reverse-recalculation and checkpointing scheme, which ensured accuracy along the calculation paths. The scheme was applied to 3D multi-channel CS-MRI, as well as super-resolution optical microscopy, showing huge reduction in memory usage during training and testing. Additionally, Zhang *et al*. [170] suggested memory-efficient learning by distributing the learning task on multi-GPUs. The memory was further leveraged by using mixed-precision processing, where the computations in CNN were processed in half precision. Another work [171] tackled the memory issue by dividing whole volume data into smaller size slabs, also using a model-based unrolled iterative scheme. The proposed data augmentation strategy overcame the GPU memory limitations, outperforming 2D processing by utilizing multi-dimensional correlations.

#### 4D data reconstruction

Both spatial and temporal redundant information can be utilized in 4D data reconstruction, although it is more challenging due to the need for higher computational capacity. A variational neural network was implemented to reconstruct rapid 4D aortic flow data, namely FlowVN [172]. The approach leveraged the computational burden by forming four banks of 3D filters in each regularization layer, with each bank performing 3D convolution operation of dedicated dimensions. The authors demonstrated that the proposed model generalized well to both retrospective and prospective applications with various acceleration rates and anatomies. Other model-based deep learning methods for the reconstruction of 4D MRI data include [173–175].

**Fig. 7:**
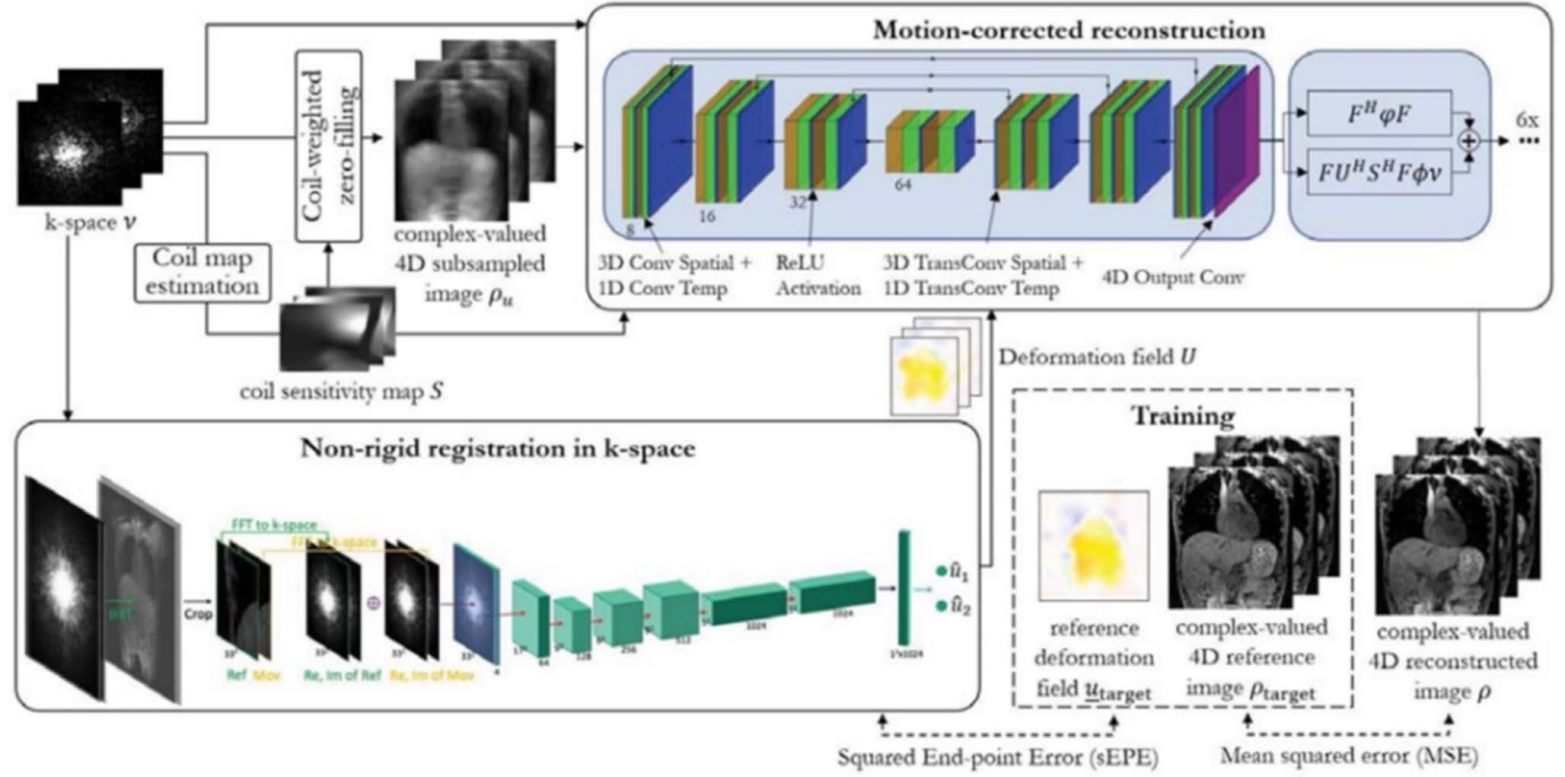
Motion-compensated 4D reconstruction network from Küstner *et al*. [176] consisting of a non-rigid registration network and a motion-corrected reconstruction network. The non-rigid registration network produces estimated deformation fields that are utilized for data consistency block of the reconstruction network. The reconstruction network utilizes coil-weighted zero-filled image $\:{\rho\:}_{u}$, coil sensitivity map $\:S$, k-space data $\:v$, and the deformation field $\:U$ to reconstruct motion-corrected image $\:\rho\:$

Another work implemented CNN-based model with (3 + 1)D convolution (Fig. 7), which operates 3D spatial convolution and 1D temporal convolution to exploit spatiotemporal redundancies from 4D body trunk data [176]. The model utilized non-rigid registration as well, enhancing the redundant information among motion states. The performance was compared with image-based registration and motion-corrected iterative SENSE reconstruction methods, with the proposed method showing superior performance. The work was followed by [177], where a self-supervised learning scheme was employed for datasets without labels. CNN-based networks were utilized in other works [178–180], which also handled various 4D data. On the other hand, Movienet was proposed for motion-preserved reconstruction of 4D abdominal MRI data [181]. The motion preservation in image domain helped reducing the reconstruction time without the nonuniform fast Fourier transform (NUFFT) operation, which was computationally burdening. The model showed comparable results to XD-GRASP [182], but with a significantly shorter reconstruction time.

## Discussion and conclusion

This paper reviewed deep learning-based accelerated MRI reconstruction methods that utilize different redundant information existing in MRI data. The correlation between MRI signals acquired by multiple coils is exploited in PI. Also, different contrast images contain similar structural information, thus are employed to reconstruct target contrast images. The advances in hardware have allowed networks to utilize spatial and temporal redundancies in high-dimensional MRI. The exploitation of MRI data redundancy has significantly affected the reconstruction performance by enhancing the preservation of structural details in the images, as well as improving robustness against perturbations.

Deep learning-based reconstruction approaches have shown great promises but there still exist challenges and limitations. One shortcoming is the data dependency of the deep learning-based methods, where a large amount of training data is required to achieve remarkable performances. This limits reconstruction in clinical applications where a large amount of data is not obtainable. Numerous works have tried to alleviate the limitation in various approaches including data augmentation [38, 183], transfer learning [184–186], unsupervised learning [162, 187, 188], self-supervised learning [80, 159, 177, 189], and so forth. Among these approaches, the self-supervised learning has high potential since the training can be done with the data itself. The self-supervised learning approaches utilize the acquired k-space data for training, not enforcing to learn the distribution of the reference data [36, 190–192]. These methods show superb performance without the acquisition of full-sampled data. Further developments in such approaches may accelerate the application of deep learning-based methods to clinical settings.

Another limitation is the interpretability of the deep neural network. More specifically, end-to-end networks are trained to learn the mapping from input to output, exhibiting a lack of interpretation. There exist works on interpretable networks based on mathematical derivations [193, 194], but the complete explanation of the “black box” in deep learning methods is still a challenge. The model-based unrolled iterative methods may provide better interpretability since it is based on the conventional CS-MRI. Such methods are derived from the widely used iterative optimization algorithms and established mathematical models for MRI reconstruction. The network architectures are dependent on the optimization algorithm, where these networks are trained with physics-based constraints, achieving better interpretability than fully data-driven models. Implementation of more explainable deep learning techniques such as attention module or integration with MRI physics or clinical knowledge may be options to compensate for this shortcoming.

The joint multi-contrast MRI reconstruction boosts the quality of reconstructed images without doubt but the performance may degrade due to a few issues. The movement during or between scans for multi-contrast imaging can be one issue. Motion artifacts during the scan may degrade the reconstruction performance, while the bulk movement between scans cause misregistration between contrasts. Previous works have dealt with these issues by processing in k-space domain [140] or using spatial transformation module [142]. Implementation of motion correction networks [195, 196] or registration networks [197, 198] may be other options. Feature leakage is another issue, where unique features from one contrast are transferred to other contrasts. Several works have tried to overcome this issue. Kopanoglu *et al*. [199] utilized simultaneous joint and individual regularization terms among the contrasts while Dar *et al*. [145] exploited low frequency prior of the target contrast to prevent feature leakage.

Despite the fact that advances in hardware have enabled utilization of deeper neural networks and larger data, computational cost is still a crucial limitation for reconstruction of high dimensional MR images [200]. Previous 3D and 4D reconstruction methods have divided the data into smaller volumes [171] or employed a sequence of smaller dimensional convolution operations [166, 176]. However, such approaches do not fully utilize spatial and temporal redundant information. Other works suggested memory-efficient learning algorithms [169, 170], sacrificing computation time as trade-off. Further developments in efficient algorithms and hardware performance, as well as novel data processing techniques that better utilize the spatiotemporal redundancy may help overcome the limitation.

In summary, redundancy exists in spatial and temporal domains of MRI data, as well as in multi-contrast MRI data. Exploiting the redundant information in various MRI data provides improved acceleration and reconstruction performance. Recent integration with deep learning approaches has achieved further improvements. While there remain limitations, utilization of redundant information combined with deep learning-based reconstruction approaches shows promising results.
